# How We Treat Primary Hyperoxaluria Type 1

**DOI:** 10.2215/CJN.0000000000000460

**Published:** 2024-03-18

**Authors:** Matthew C. Breeggemann, Peter C. Harris, John C. Lieske, Gregory E. Tasian, Kyle D. Wood

**Affiliations:** 1 Division of Nephrology, University of California San Francisco, San Francisco, California; 2 Division of Nephrology and Hypertension, Mayo Clinic, Rochester, Minnesota; 3 Department of Laboratory Medicine, Mayo Clinic, Rochester, Minnesota; 4 Division of Urology, Department of Surgery, The Children's Hospital of Philadelphia, Philadelphia, Pennsylvania; 5 Department of Biostatistics, Epidemiology, and Informatics, University of Pennsylvania, Philadelphia, Pennsylvania; 6 Department of Urology, University of Alabama at Birmingham, Birmingham, Alabama

**Keywords:** CKD, genetic renal disease, kidney stones

## Introduction

Primary hyperoxaluria type 1 (*AGXT*), type 2 (*GRHPR*), and type 3 (*HOGA1*) represent a family of rare, autosomal recessive disorders characterized by excessive hepatic production and excretion of oxalate by the kidneys.^1^ Disease sequelae include kidney stone disease, nephrocalcinosis, and progressive CKD. Kidney failure and systemic oxalosis can occur if the disease is not diagnosed and/or left untreated.^1^ As signs and symptoms of primary hyperoxaluria overlap with other kidney stone diseases, a diagnosis cannot be resolved by stone analysis and imaging alone. Significant progress in targeted primary hyperoxaluria therapeutics has increased the importance of an early diagnosis. Greater availability of genetic testing has accompanied these treatment advances. Here, we emphasize the utility of genetic testing in routine diagnostic evaluation of patients suspected of having primary hyperoxaluria and at-risk family members, which also applies to the assessment of patients with other suspected monogenic kidney stone diseases.

## Two Cases: Different Journeys

Adult patient: A 42-year-old woman presented with CKD stage 3B (eGFR, 43 ml/min per 1.73 m^2^), followed by rapid deterioration, ultimately requiring hemodialysis. Medical history included a first kidney stone event at age 18 months, kidney stone–induced flank pain necessitating emergency room visits throughout adolescence and adulthood, and numerous and wide-ranging surgical procedures to manage significant stone burden. A kidney biopsy demonstrating oxalate nephropathy led to consultation with an academic medical center, genetic testing, and metabolic workup (predialysis 24-hour urinary oxalate [Uox] excretion, 1.40 and 1.23 mmol/1.73 m^2^; adult reference range, <0.46 mmol/1.73 m^2^: plasma oxalate [Pox], 82 *µ*mol/L; adult reference range, ≤2.0 *µ*mol/L) and ultimately a primary hyperoxaluria type 1 diagnosis (homozygous *AGXT* p.Gly170Arg). While maintaining dialysis, concomitant therapy with pyridoxine (vitamin B6)^1^ and the small interfering RNA lumasiran^2^ resulted in a 24-hour Uox of 0.64 mmol/1.73 m^2^ (<1.5×upper limit of normal) and a Pox of 14 *µ*mol/L (*i.e*., an 83% drop) after 6 months of treatment. Pyridoxine is an essential liver-peroxisomal alanine:glyoxylate aminotransferase cofactor that can stabilize specific mistargeting pathogenic variants (*i.e*., p.Gly170Arg, p.Phe152Ile, and p.Ile244Thr).^1,2^ Lumasiran targets glycolate oxidase, an enzyme upstream of alanine:glyoxylate aminotransferase, lowering Uox levels^2^ and possibly allowing for a future kidney-only transplant. The approved small interfering RNA nedosiran also lowers Uox levels by targeting the downstream hepatic lactate dehydrogenase enzyme,^2^ which catalyzes the conversion of glyoxylate to oxalate, and represents another treatment option for suitable PH1 patients.

Hypothetical childhood patient for comparison: A previously healthy 3-year-old boy was referred to Urology because of hematuria. A kidney bladder ultrasound detects bilateral stones, nephrocalcinosis, and hydronephrosis. He is not yet toilet trained; a spot urine test reveals an oxalate-to-creatinine ratio of 200 *μ*mol/mmol (reference range 2−5 years, 19−101 *μ*mol/mmol).^2^ He has preserved kidney function with an eGFR of 95 ml/min per 1.73 m^2^ by the CKD in Children Under 25-equation. Genetic testing reveals primary hyperoxaluria type 1 (homozygous p.Ile244Thr) and treatment with lumasiran is initiated. Response to lumasiran will be assessed by monthly spot urine tests^2^ until the child can accurately provide a 24-hour urine collection. He will have bilateral percutaneous nephrolithotomies to remove the kidney stones, and will receive a kidney ultrasound every 3 months to evaluate stone recurrence.

## Evaluation

**Figure 1:**
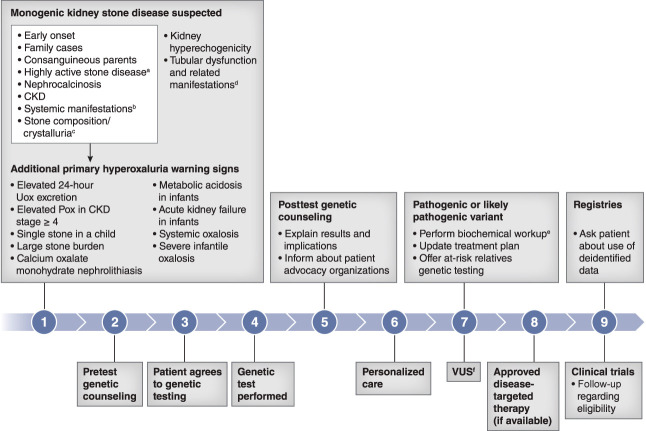
**Investigation of a patient suspected of monogenic kidney stone disease.** ^a^Bilateral, multiple stones, frequently recurrent. ^b^Sensorineural hearing defects, ocular abnormalities, neurological disorders. ^c^Monohydrate calcium oxalate (whewellite), cystine, dihydroxyadenine, xanthine. ^d^Statural growth deficit, polyuria, bone disorders. ^e^Enables characterization of clinical status and guides treatment decisions and monitoring. ^f^Avoid making clinical decisions on the basis of VUS, perform biochemical and possibly additional genetic tests to aid diagnosis, periodic re-evaluation if the patient so chooses under the guidance of a genetic counselor. Pox, plasma oxalate; Uox, urinary oxalate; VUS, variant of uncertain significance.

Several broad genetic screening studies of patients with suspected monogenic kidney stone have yielded a genetic diagnosis rate of approximately 10% in adult and approximately 20% in pediatric populations.^3^ Several factors strongly associated with monogenic kidney stone disease suggest considering genetic testing, including a kidney stone before age 25 years, recurrent bilateral stones, nephrocalcinosis, known family history of recurrent kidney stones, and/or CKD without an underlying etiology (Figure 1). Recognizing primary hyperoxaluria is challenging because of its rarity and clinical heterogeneity,^1^ especially when there is no known family history.

## Diagnostic Critique

Genetic testing is standard of care whenever primary hyperoxaluria is suspected.^2^ Typically, the key biochemical investigations are 24-hour Uox excretion or Pox if the patient has CKD stage 4 or 5 (per OxalEurope/ERKNet recommendations).^2^ The American Urological Association recommends genetic testing if 24-hour Uox excretion exceeds 0.83 mmol/1.73 m^2^ (75 mg/1.73 m^2^).^4^ However, these guidelines do not capture all patients for whom there is a high clinical suspicion for primary hyperoxaluria such as the commonly seen pediatric patient described above, with large, bilateral stones and nephrocalcinosis, an inability to provide a 24-hour urine collection, and normal eGFR.^5^ The wide variability in spot urine data means repeating the test several times to confirm positive or negative results if it is the only testing possible in younger patients. Even in older children and adults, 24-hour urine testing has been underused by providers, and patients may not accurately collect timed urine samples, resulting in missed diagnoses.^6^ Thus, in a real-world setting, parallel genetic assessment and urinalysis are recommended as a more efficient and reliable means to reach a diagnosis employing complementary results. Furthermore, preemptive genetic screening in patients with risk factors for monogenic kidney stone disease can narrow the biochemical workup and avoid overly extensive biochemical testing.

Genetic testing for suspected monogenic kidney stone disease is not routinely performed in most clinical settings. In our adult case, despite multiple touchpoints with health care providers, clues of an underlying genetic condition went unrecognized. Lack of detailed metabolic data earlier in the clinical course also limited suspicion of primary hyperoxaluria. The delay in genetic testing was likely due to the fragmented care that she received from rural hospitals throughout her life. Earlier referral to a tertiary center with kidney stone disease and genetic expertise could have been beneficial, as would administration of the recently developed clinical screening instrument for the diagnosis of primary hyperoxaluria type 1 in adult patients undergoing dialysis.^7^

## Genetic Testing

Genetic testing can rapidly provide an accurate diagnosis, enable initiation of disease-specific treatment (potentially *via* enrollment in clinical trials/registries), and inform on inheritance and carrier risk (Figure 1). Genetic test results provide information on the disease manifestation and potential severity of outcomes, the gene involved, and the detected variants. Variants are classified in a five-tiered scheme as pathogenic, likely pathogenic, likely benign, benign, or variant of uncertain significance (VUS) on the basis of the weighted quantity and quality of evidence; only pathogenic and likely pathogenic results indicate a positive diagnosis.^8^

Panels containing the approximately 40 known monogenic kidney stone disease genes or ones more narrowly focused on the three primary hyperoxaluria genes are used. The *AGXT* mutations observed in our adult (p.Gly170Arg) and pediatric cases (p.Ile244Thr) account for approximately 30% and 6% of primary hyperoxaluria type 1 alleles,^9^ respectively, with corresponding median ages for homozygotes at kidney failure of 47 and 33 years without targeted therapy.^9,10^

Since rigorous criteria categorize a variant to a pathogenic or benign group,^8^ a significant number of variants are defined as VUS. A VUS is challenging for both patients and clinicians since, by definition, no clinical decisions can be made based solely on these results. However, specific follow-up investigations (*e.g*., familial segregation analysis, primary hyperoxaluria urine metabolites [glycolate, L-glycerate, 4-hydroxy-2-oxoglutarate, and 2,4-dihydroxyglutarate], functional testing, genomic sequencing) can sometimes resolve the VUS to a diagnostic category.

Genetic testing is an important diagnostic tool early in the evaluation of patients presenting with cardinal signs of primary hyperoxaluria and other individuals with recurrent kidney stone disease, nephrocalcinosis, and/or CKD. If the test provides a molecular diagnosis, valuable prognostic information is conferred and an updated personalized treatment plan is developed.
